# Multisensory integration in humans with spinal cord injury

**DOI:** 10.1038/s41598-022-26678-x

**Published:** 2022-12-22

**Authors:** Roberta Vastano, Marcello Costantini, William H. Alexander, Eva Widerstrom-Noga

**Affiliations:** 1 Department of Neurological Surgery, The Miami Project to Cure Paralysis, University of Miami, Miami, FL 33136 USA; 2 Department of Psychological, Health and Territorial Sciences, “G. d’Annunzio” University of Chieti-Pescara, Chieti, Italy; 3 Institute for Advanced Biomedical Technologies, ITAB, “G. d’Annunzio” University of Chieti-Pescara, Chieti, Italy; 4 Center for Complex Systems and Brain Sciences, Florida Atlantic University, Boca Raton, USA; 5 Department of Psychology, Florida Atlantic University, Boca Raton, USA; 6 The Brain Institute, Florida Atlantic University, Boca Raton, USA

**Keywords:** Cognitive neuroscience, Computational neuroscience, Sensory processing, Spinal cord

## Abstract

Although multisensory integration (MSI) has been extensively studied, the underlying mechanisms remain a topic of ongoing debate. Here we investigate these mechanisms by comparing MSI in healthy controls to a clinical population with spinal cord injury (SCI). Deafferentation following SCI induces sensorimotor impairment, which may alter the ability to synthesize cross-modal information. We applied mathematical and computational modeling to reaction time data recorded in response to temporally congruent cross-modal stimuli. We found that MSI in both SCI and healthy controls is best explained by cross-modal perceptual competition, highlighting a common competition mechanism. Relative to controls, MSI impairments in SCI participants were better explained by reduced stimulus salience leading to increased cross-modal competition. By combining traditional analyses with model-based approaches, we examine how MSI is realized during normal function, and how it is compromised in a clinical population. Our findings support future investigations identifying and rehabilitating MSI deficits in clinical disorders.

## Introduction

Our perception of the environment is often a result of a multisensory integration (MSI), defined as the ability to synthesize cross-modal sensory information within temporal and spatial contingencies, reduce environmental uncertainty and facilitate both detection and action^1,2^. This facilitatory effect is known as *multisensory enhancement*^3,4^. Enhancement refers to improvements in behavioral performance (e.g., reaction times and accuracy), as well as stronger neural responses across a large network involving subcortical structures, like thalamus^5–8^ and superior colliculus^1^, sensory-specific^6,9^ and non-sensory specific multisensory areas^3,10,11^.

Animal^1–3,12^ and human^3,13–15^ studies have shown that that cross-modal stimuli induce a response enhancement that is generally super-additive, i.e., the multimodal response (both at behavioral and neural level) is greater than the summed unimodal responses^4^. However, in certain circumstances MSI may be reduced, as no integration, sub-additive (i.e., the multimodal response is less than the summed unimodal responses), or even depression effects can arise due to factors like sensory impairments, abnormal perceptual abilities, and functional abnormality in associative areas. For instance, schizophrenia patients have reduced MSI linked to impaired ability to filter redundant sensory information, auditory and/or visual hallucinations^16,17^. Individuals with hearing and vision loss^9,18–20^ show reduced audio-visual integration related to reorganization of visual cortical areas and inadequate visual processing. Finally, eating disorders (e.g., anorexia nervosa (AN^21,22^)) and autism spectrum disorder (ASD)^23–25^ are also associated with altered MSI and asymmetric interhemispheric theta power with lower power in the right centro-parietal areas, and impaired temporal processing, respectively.

Although deficits in MSI have been documented across a number of disorders^9,16,18–20,22,24,26^, the influence of SCI on MSI remains incompletely known. Both anatomical^27,28^ and functional^28–31^ changes in primary motor and somatosensory cortices have been observed in individuals with SCI. Among several mechanisms underlying such reorganization, research supports the idea that compensatory use of a less affected body part facilitates axonal sprouting towards the area of the deafferented body part^31,32^, long-term potentiation mechanisms^32^, and unmasking of pre-existing lateral dormant connections due to decreased GABAergic inhibition after injury^33–36^.

Recently we proposed^37^ that the consequences of such reorganization, together with sensorimotor impairments in SCI^31,38^, may induce multisensory depression and/or competition mechanisms that oppose a multisensory enhancement effect. These effects may be caused by unisensory imbalance in which either the intact visual or the auditory system dominates over impaired somatosensory and proprioceptive systems and therefore reduce the integration of cross-modal information from sensory and non-sensory specific multisensory areas. Congruent with this assumption, animal research^39^ has shown response depression to cross-modal stimuli (e.g., audio-visual) in animals reared in the dark: to reduce noise, relevant unisensory signals are preferred over the ones that appear to be weak, and a default competition mechanism depresses neural responses to weaker cross-modal signals. Evidence from humans supports a similar view: multisensory responses are fastest when the intensity of unisensory stimuli are balanced and participants show similar performance for unisensory stimuli^40^.

Additionally, given the evidence suggesting that the functional integrity of the perceiver’s sensory^9,20^ and motor^41,42^ systems affects MSI, it is likely that the sensorimotor deficits caused by SCI impact MSI. During integration, stimuli compete not only because of unisensory imbalance^39,40^, but also for access to the motor system^3,43^. Responses to cross-modal stimuli are generally super-additive within a brief time-window^44–47^ following stimulus onset. Behavioral studies in humans also show that super-additive effects of MSI are usually observed and analyzed within a time-window including only the fastest reaction times^48,49^. In conditions affecting the motor system, as in SCI, it is possible that the motor command is interrupted or is present but cannot be quickly initiated. Thus, motor impairment may leave more time for stimuli to compete and generate a multisensory depression.

Intact exteroceptive multisensory information processing is considered to play a pivotal role in many, if not most, cognitive operations^50^. Consequently, alterations in such processing may have detrimental effects on cognitive functions. Importantly, depersonalization, phantom pain and other phantom sensations^51^, often observed in SCI patients, have been considered to originate from altered processing of multisensory information. Hence, understanding MSI after SCI might have important implications for new therapeutic avenues in SCI patients.

The goals of this study were twofold. First, we aimed to compare behavioral performance during MSI in uninjured controls and in SCI population to determine the extent to which MSI is compromised after SCI. To this end we used traditional statistical tests, such as the independent race model^52–54^. To the best of our knowledge, this is the first study to evaluate MSI in this clinical population. Second, we adopted a model-based approach to investigate two hypotheses regarding how SCI might contribute to MSI deficits. One possibility is that SCI may lead to an increase in unisensory imbalance between sensory modalities impaired by SCI (e.g., somatosensory) and modalities that are unaffected by SCI (e.g., visual and auditory). Alternatively, MSI deficits may be due to increased competition for access to the motor system across all sensory modalities arising from the SCI-related motor impairment.

Data were collected using a standard detection task in which participants (healthy controls and individuals with SCI) were instructed to respond to a target stimulus as quickly as possible. Targets were presented in three sensory modalities (visual, auditory, tactile). In a unimodal condition, targets were presented from a single modality, while in a bimodal condition, redundant targets were presented (temporally congruent) from two of the three modalities (audio-visual, visuo-tactile, audio-tactile). To overcome somatosensory and motor deficits in SCI individuals, we delivered the tactile stimulus above level of injury and recorded vocal reaction times (RTs) for all participants.

To analyze our data, we used the independent race model and model-based approaches. The independent race model^52–54^ for RTs data is frequently used to quantify the multisensory enhancement effect. This allows us to dissociate MSI from bimodal facilitation by redundant unimodal stimuli. The multisensory enhancement effect is expressed as a *violation* of the race model, in which RTs for multisensory stimuli are *faster* than the summed unisensory probabilities. Conversely, deficits in MSI are indicated by conditions in which RTs for multisensory stimuli are *slower* than the summed unisensory probabilities.

To test whether MSI deficits in SCI are the product of unisensory imbalance or increased competition, we compared two biologically inspired computational models of information accumulation^55,56^. This approach allowed us to determine whether MSI differences in SCI (compared to healthy controls) were associated with changes in sensory processing pathways caused by neural reorganization following injury, and at which point in the processing stream (e.g., the level of stimulus or perceptual representation) these changes occur. Even in the case that sensory cortical processing in those with SCI is structurally similar to healthy controls, we hypothesized that behavioral differences may be due to *a)* alterations in the salience of stimuli in one or more modalities, *b)* increased cross-modal competition at either the stimulus or perceptual level, or *c)* a general increase in processing noise.

## Results

### Raw RTs results

**Figure 1:**
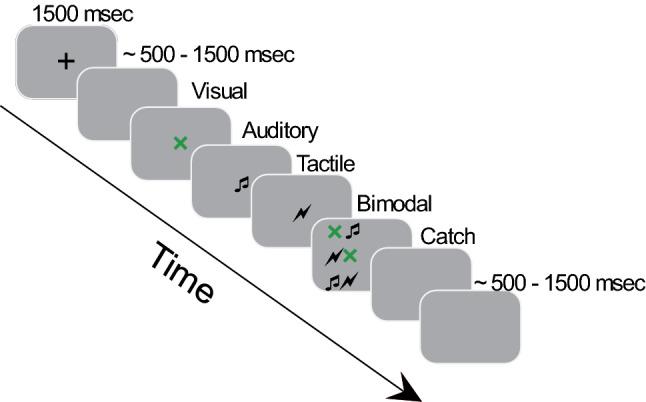
Timeline of the experimental trials: Each trial started with a fixation cross (1500 ms) followed after a variable blank screen (~ 500–1500 ms) by *one* of the following stimuli for which participant had to respond vocally: Visual (green X), Auditory (a pure tone 1000 Hz), Tactile (a suprathreshold electrical stimulus delivered on the forehead), bimodal (Audio-visual (AV) or Visuo-tactile (VT) or Audio-tactile (AT)). In the 20% of the trials no stimulation was delivered (Catch). Finally, there was a variable intertrial interval (ITI) (~ 500–1500 ms) before next trial started.

**Figure 2:**
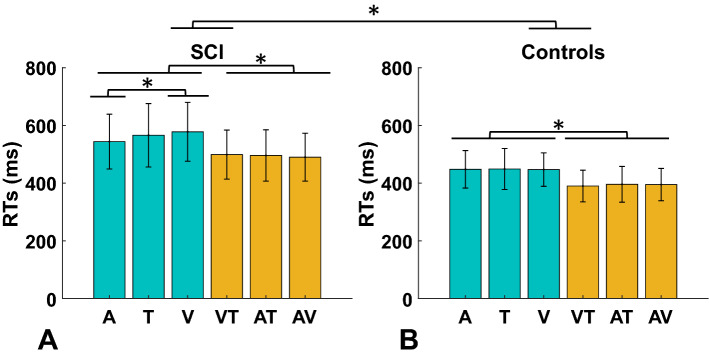
Reaction Times** (RTs**) of SCI participants (**A**) and healthy controls (**B**)**:** Green bars show the unimodal stimuli (Audio, Tactile and Visual), yellow bars show the bimodal stimuli (Visuo-tactile, audio-tactile, and audiovisual). (Error bars indicate the standard deviation; *p < 0.05).

We recorded vocal reaction times (RTs) during a detection task in which redundant targets were unimodal (visual (V), auditory (A), tactile (T)) and bimodal (audio-visual, visuo-tactile, audio-tactile) (Fig. 1). We analyzed raw RTs to verify the presence of the redundant signals effect (RSE) and investigate differences between groups and stimulus modalities. To this end, three separate 2 × 3 between-subjects ANOVAs were performed with Group (SCI/healthy controls) as a categorial factor and stimulus-modality (A/V/AV); (V/ T/VT) or (A/T/AT) as dependent variables. RT results showed that bimodal stimuli were faster than unimodal stimuli, confirming the RSE in both groups. However, in general, SCI participants tended to be slower than healthy controls in all conditions. In fact, results from between-subjects ANOVAs in A/V/AV stimulus-modality showed a significant main effect of the group: (F (1,30) = 16.17; *p* < 0.001; η_p_^2^ = 0.35), stimulus-modality (F (2,60) = 66.52; *p* < 0.001; η_p_^2^ = 0.68) and their interaction (F (2,60) = 5.01; *p* = 0.009; η_p_^2^ = 0.14). Newman-Keuls posthoc indicated that RTs in healthy controls were faster compared to SCI participants (A stimuli healthy controls: Mean = 448 ms, SD = 65 ms; A stimuli SCI: Mean = 544 ms, SD = 95 ms; *p* = 0.003; V stimuli healthy controls: Mean = 447 ms, SD = 71 ms; V stimuli SCI: Mean = 577 ms, SD = 102 ms; *p* < 0.001; AV stimuli healthy controls: Mean = 395 ms, SD = 62 ms; AV stimuli SCI: Mean = 490 ms, SD = 83 ms; *p* = 0.008) (Fig. 2).

In addition, there was no difference in the RTs between the unimodal conditions in the control group (A: Mean = 448 ms, SD = 65 ms vs. V: Mean = 447 ms, SD = 58 ms, *p* = 0.93). However, SCI participants were faster in the A condition (Mean = 544 ms, SD = 95 ms) compared with V (Mean = 577 ms, SD = 102 ms) (*p* < 0.001). (Fig. 2A).

Results from V/T/VT stimulus-modality showed a significant main effect of the group: (F (1,30) = 17.75; *p* < 0.001; η_p_^2^ = 0.37) and stimulus-modality (F (2,60) = 71.87; *p* < 0.001; η_p_^2^ = 0.7). Newman-Keuls posthoc indicated that RTs in healthy controls were overall faster compared with SCI participants (healthy controls: Mean = 429 ms, SD = 67 ms; SCI: Mean = 547 ms, SD = 103 ms; *p* < 0.001). RTs in VT condition were faster (Mean = 441 ms, SD = 88 ms) compared to the correspondent unimodal (V: Mean = 508 ms, SD = 104 ms, *p* < 0.001; T: Mean = 503 ms, SD = 107 ms, *p* < 0.001). No interaction between groups and stimulus-modality was observed (F (2,60) = 1.45; *p* = 0.241; η_p_^2^ = 0.04). (Fig. 2).

Results from A/T/AT stimulus-modality showed a significant main effect of the group: (F (1,30) = 13.63; *p* < 0.001; η_p_^2^ = 0.31) and stimulus-modality (F (2,60) = 36.19; *p* < 0.001; η_p_^2^ = 0.54). Newman-Keuls posthoc indicated that RTs in healthy controls were overall faster compared to SCI participants (healthy controls: Mean = 431 ms, SD = 69 ms; SCI: Mean = 535 ms, SD = 100 ms; *p* = 0.001). RTs were faster in AT (Mean = 443 ms, SD = 90 ms) condition compared with the correspondent unimodal (T: Mean = 503 ms, SD = 107 ms, *p* < 0.001; A: Mean = 493 ms, SD = 93 ms, *p* < 0.001). No interaction between groups and stimulus-modality was observed (F (2,60) = 0.98; *p* = 0.378; η_p_^2^ = 0.03). (Fig. 2).

### Independent race model results: Gondan’s permutation test

**Figure 3:**
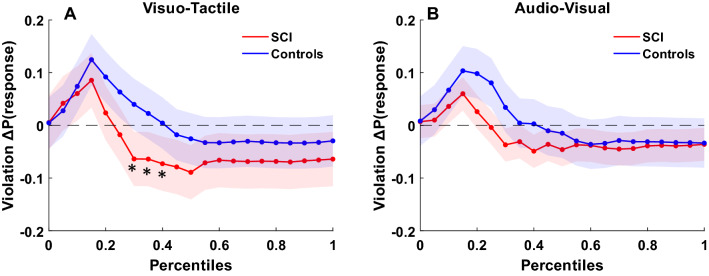
Race model violations (for the fastest RTs (40%)): Figure shows violations in Visuo-Tactile (**A**) and Audio-Visual (**B**) conditions for each group (red lines represent SCI participants, blue lines healthy controls) measured as the difference (Δ) in the CDFs between bimodal and the correspondent race (known as Miller’s Inequality). Positive values (above 0) denote the multisensory enhancement effect. Color shade indicates standard deviation. (*p < 0.05).

To investigate MSI effects, violations of the race model were tested using Gondan’s permutation test over the fastest 40% of responses. This value was chosen based on a visual inspection of the Miller’s inequality plots (i.e., differences between bimodal and race model CDFs) in healthy controls. For our control group, violation of the race model was observed in all the conditions (AV: tmax = 5.1, tcrit = 2.4, *p* = 0.001; AT: tmax = 3.7, tcrit = 2.3, *p* = 0.003; VT: tmax = 5.3, tcrit = 2.2, *p* < 0.001). In contrast, SCI participants exhibited violation of the race model only in AV and VT conditions (AV: tmax = 2.6, tcrit = 2.5, *p* = 0.037; AT: tmax = 1.7, tcrit = 2.4, *p* = 0.16; VT: tmax = 3.6, tcrit = 2.5, *p* = 0.008). Hence the AT condition was not further considered for comparisons between groups. To compare the magnitude of MSI across SCI participants and healthy controls, simple effect analyses were performed. For the AV condition, no simple effect analyses turned out to be significant, suggesting a comparable magnitude of MSI in healthy controls and SCI participants (Fig. 3A). For the VT condition, results revealed a higher magnitude (i.e., more efficient) of MSI in healthy controls as compared to SCI participants in the 7th, 8th and 9th bins corresponding to the 30%, 35% and 40% of fastest responses (Fig. 3B) (7th bin: t(30) = 2.8, *p* = 0.009, Cohen’s d = 0.99; 8th bin: t(30) = 2.5, *p* = 0.016, Cohen’s d = 0.90; 9th bin: t(30) = 2.9, *p* = 0.007, Cohen’s d = 1.03).

Overall, these results confirm that MSI in SCI participants is impaired to a greater extent in the audio-tactile modality, followed by visuo-tactile. In contrast, the audio-visual modality showed similar integration effects between groups. These results are congruent with our hypotheses of unisensory imbalance and indicate alteration in sensory processing following SCI.

### Independent race model results: logistic fit

**Figure 4:**
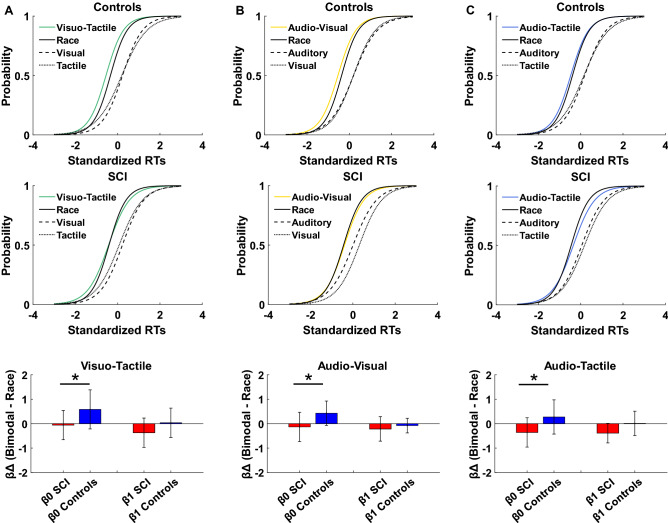
Logistic fit of the CDFs in healthy controls and SCI participants: Figure shows logistic fit in bimodal conditions (**A**): Visuo-Tactile: green line; (**B**): Audio-Visual: yellow line and (**C**): Audio-Tactile: light blue line) and race model (Race: black line). Unimodal conditions are represented by black dashed lines. Standardized RTs indicate z-scores. Bar plots (below each logistic fit and stimulus modality) indicate the difference (Δ) between bimodal and the correspondent race of the coefficients (β0: intercept and β1: slope expressed as log (odds)) in sci (red bars) and control subjects (blue bars). (Error bars indicate standard deviation).

**Figure 5:**
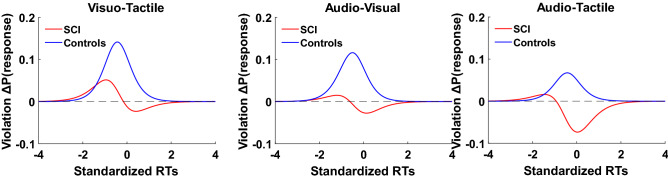
Race model violations (for the entire RT distribution): Figure shows violations in each condition for each group (red lines represent SCI participants, blue lines healthy controls) measured as the difference (Δ) in the CDFs between bimodal and the correspondent race (known as Miller’s Inequality). Positive values (above 0) denote the multisensory enhancement effect. Standardized RTs indicate z-scores.

In the previous analysis, only a subset of the RT data for the fastest trials was used for analysis. Although this approach is standard in studies of MSI, it is usually the case that the race model is fitted to the entire distribution of RTs^57^. To verify that our results were not due only to conducting our analyses on a subset of data, we therefore conducted a second analysis with the full distribution of RTs for SCI and healthy controls. For each subject, we fitted a logistic model to the RT distribution of standardized RTs for each bimodal condition as well as the RT distribution predicted by the independent race model. For each fit, we obtained two coefficients, β0 and β1, indicating the intercept and slope of the best-fit logistic function. Differences of the coefficients between bimodal conditions and the corresponding race were obtained for each subject and entered into a between-subject ANOVA to test race model violations. The results of a 2 × 2 × 3 (group/logistic fit coefficients/stimulus modalities: differences between race and the corresponding bimodal conditions) between-subjects ANOVA showed a significant main effect of the group (F (1,30) = 9.99; *p* = 0.003; η_p_^2^ = 0.24); coefficients (F (1,30) = 21.06; *p* < 0.001; η_p_^2^ = 0.41) and their interaction (F (1,30) = 5.59; *p* = 0.02; η_p_^2^ = 0.15). Newman-Keuls posthoc showed that differences in β0 across stimulus-modalities were lower in SCI participants (Mean = -0.18, SD = 0.6) compared to healthy controls (Mean = 0.42, SD = 0.7) (*p* = 0.001). *Positive values of β0 indicate violation of race model, while negative values indicate no violation of the model*. Different from the observations regarding the fastest RTs, the results of the entire RTs distribution suggest that regardless of stimulus-modality, participants with SCI have no to limited integration effects. This might be because individuals with SCI exhibited larger competition effects at the slower RTs. (see Fig. 4A, B and C for the logistic plots and the corresponding bar plots of β0 and β1 values in each stimulus-modality between groups. Figure 5 shows the Miller’s inequality plots). There was no significant main effect of stimulus-modality (F (2,60) = 1.13; *p* = 0.32; η_p_^2^ = 0.03), no significant interaction between stimulus-modality and group (F (2,60) = 0.39; *p* = 0.67; η_p_^2^ = 0.01), nor a significant interaction between stimulus-modality, coefficients, and group (F (2,60) = 1.43; *p* = 0.64; η_p_^2^ = 0.01). Overall, these results suggest that MSI effects in control subjects persist beyond the fastest RTs distribution, while in SCI participants MSI seems to decay with increased RTs, as we did not observe integration effects in this group. Interestingly, the same qualitative pattern of early enhancement with later attenuation is observed in all stimulus-modalities. Thus, in SCI participants the inability to initiate a quick response influences the way multisensory stimuli are integrated over time. It could be that the multimodal perception of individuals with SCI is facilitated and can result in an enhancement effect only in a short timeframe.

To evaluate possible differences based on stimulus modality, we also compared multisensory effects within the groups. The 2 × 3 (logistic fit coefficients/stimulus modalities: differences between race and the corresponding bimodal conditions) within-subjects ANOVA in the SCI group showed no significant effect of coefficients (β0-β1) (F (1,14) = 1.74; *p* = 0.2; ηp2 = 0.11) or stimulus-modality (F (2,28) = 0.66; *p* = 0.52; ηp2 = 0.04), nor their interaction (F (2,28) = 1.35; *p* = 0.3; ηp2 = 0.08). In healthy controls we observed a significant main effect of coefficients (β0-β1) (F (1,16) = 36.6; *p* < 0.001; η_p_^2^ = 0.69), but no significant effect of the stimulus-modality (F (2,32) = 0.88; *p* = 0.42; η_p_^2^ = 0.05), or their interaction (F (2,32) = 2.06; *p* = 0.14; η_p_^2^ = 0.11). Overall, the results from within-subjects ANOVA confirmed that the MSI effect, as measured over the entire RT distribution for the three stimulus-modalities, was similar within groups.

### Computational modeling results

**Figure 6:**
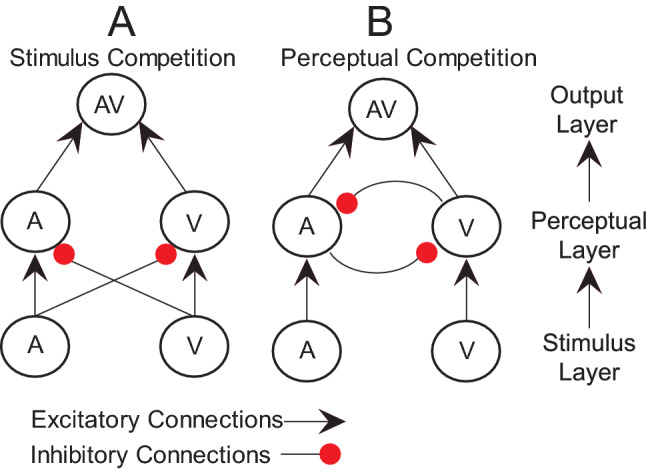
A schematic representation of the computational models of MSI: the model is organized in three layers: stimulus, perceptual and output with excitatory connections (black arrows) or inhibitory connections (red dots) The primary difference between models is whether (**A**) stimuli directly suppress perceptual representation of cross-modal stimuli or (**B**) perceptual representations compete with one another.

Our race model results indicate that, after controlling for overall differences in RTs, MSI in the SCI group is reduced relative to controls. This effect was observed for fast trials (up to 40% of the RT distribution), while for slow RTs, SCI participants showed sub-additive effects only. However, based on these results, it remains unclear *why* SCI negatively influences MSI. One possible reason underlying these differences may relate to sensorimotor deficits and structural changes following SCI (e.g., reorganization of the sensorimotor cortex^27,28^) that impact early sensory processing, integration, or response generation. To assess this, we implemented two models of information accumulation derived from the literature^58^. In information accumulation models, perception is a function of information accumulated from multiple sensory stimuli: sensory input provides evidence that is accumulated at the perceptual level to inform behavior. Competition between stimuli can occur either through feed-forward inhibition mechanisms^55^ (here called Stimulus Competition, Fig. 6A) or through lateral inhibition between accumulators (Perceptual Competition, Fig. 6B)^56^. Evidence for both forms of competition has been observed in perceptual choice studies^59^.

Here, we tested whether MSI differences after SCI derive from changes in the structure of inhibition in information processing. To assess this, we compared the average mean squared difference between fits of our Stimulus Competition and Perceptual Competition models. Summarizing our race model analyses, we conducted two fits of our models, one in which we performed a median split on our RT data and used only the fastest trials, and another using the entire RT distribution from each subject. For the first model fit (fastest trials), a 3-way ANOVA (2 × 2x3; Model Type/Group/Sensory Condition) produced a main effect of Model Type (the Perceptual Competition model fit better than Stimulus Competition, F = 29.92, *p* < 0.001, η_p_^2^ = 0.143). No other main effects or interactions were significant. For the second model fit (all trials) a 3-way ANOVA (2 × 2x3; Model Type/Group/Sensory Condition) showed a main effect of Group (model fits overall were worse for SCI than Control, F = 13.54, *p* = 0.0003, η_p_^2^ = 0.69) and a main effect of Model Type (Fits of the Perceptual Competition model were better than the Stimulus Competition; F = 22.64, *p* < 0.001, η_p_^2^ = 0.11). No other effects, including the Group by Model interaction that would indicate that the groups are better described by different models, were significant. Based on the results of these tests, we conclude that behavior for both SCI and healthy controls was a better fit by the Perceptual Competition model.

Given that both groups’ RT data were better described by the same Perceptual Competition model, we further questioned whether differences between groups might derive from changes in stimulus salience, competition between perceptual representations of sensory stimuli, or whether these differences might be due to a general deficit in processing sensory stimuli (processing noise). We evaluated this by testing differences in best-fit parameter estimates for the Perceptual Competition model with a 3-way ANOVA (2 × 3x7; Group/Condition/Parameter). As before, these tests were conducted on model fits to the fast trials as well as all trials.

**Figure 7:**
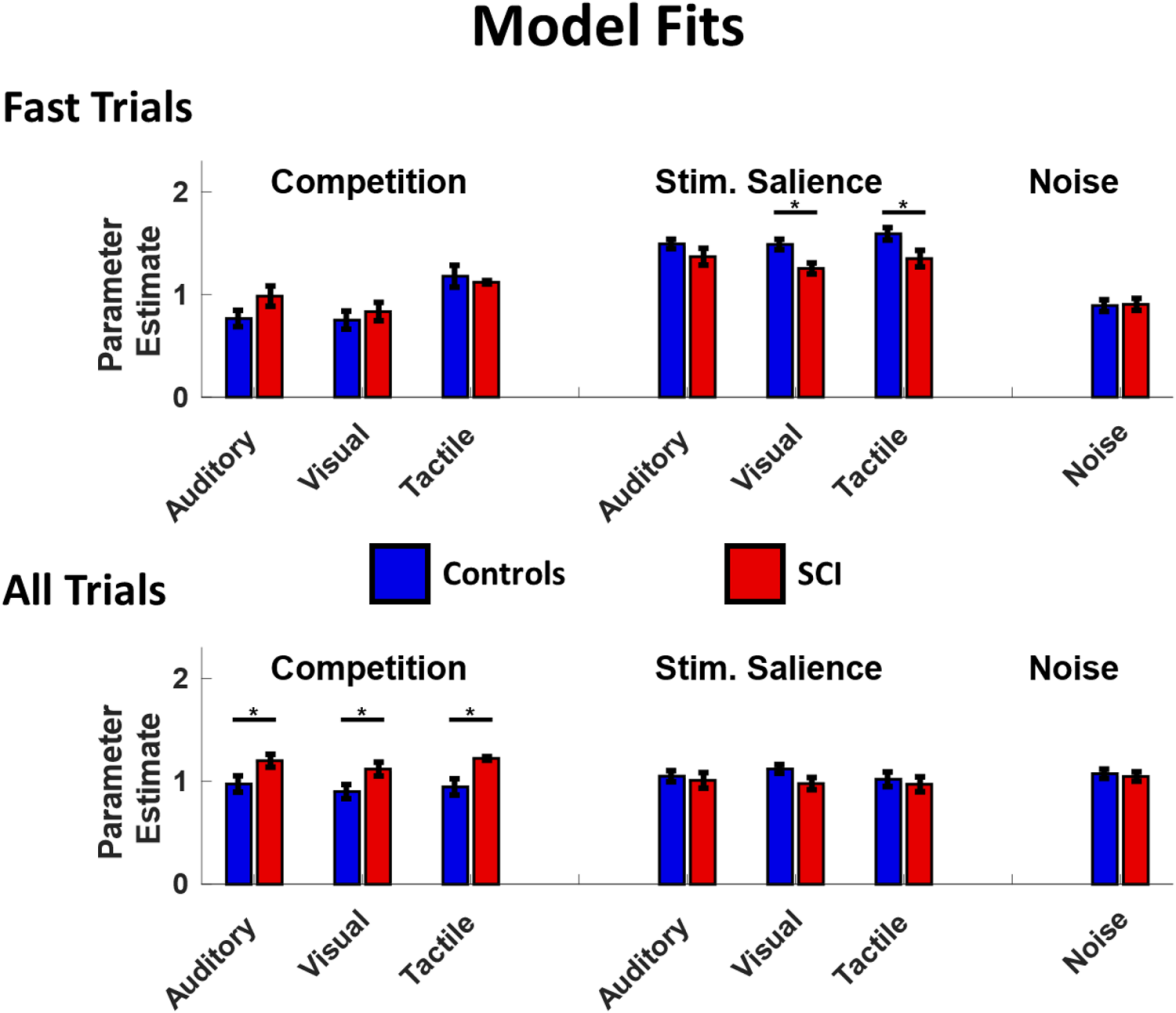
Parameter estimate: Figure shows parameter estimate for both fast trials (panel above) and slow trials (panel below) in each of the model tested (competition, stimulus salience and noise). Blue bars indicate control subjects and red bars indicate subjects with SCI. (Error bars indicate the standard error of the mean; *p < 0.05).

For the fast trials ANOVA, we observed a main effect of parameter values (F = 63.41, *p* < 0.001, η_p_^2^ = 0.458) and an interaction of Group by Parameter (F = 5.75, *p* < 0.001, η_p_^2^ = 0.071). The main effect of parameter values is unimportant in this context since, by design, the parameters are expected to have different values. No other effects or interactions were significant. Posthoc comparisons (Bonferroni corrected) showed that the Group by Parameter interaction was driven by higher stimulus salience in healthy controls for the visual (*p* = 0.001, CI = 0.10–0.404) and tactile (*p* = 0.004, CI = 0.071–0.375) modalities, while there was no difference in auditory salience (*p* = 0.108, CI = − 0.028–0.276) (Fig. 7).

For the ANOVA using all trials, we found a main effect of group (F = 7.07, *p* = 0.008, η_p_^2^ = 0.015), and an interaction of Group by Parameter (F = 6.54, *p* < 0.001, η_p_^2^ = 0.080). Post-hoc comparisons showed that, generally, perceptual competition parameters were higher for SCI relative to healthy controls (Auditory competition: Mean Difference = 0.226, *p* < 0.001, CI = 0.096–0.356; Visual competition: Mean Difference = 0.272, *p* < 0.001, CI = 0.142–0.401, Tactile Competition: Mean Difference = 0.224, *p* < 0.001, CI = 0.094–0.354 all comparisons Bonferroni corrected). There was no difference between groups for all other parameters (Fig. 7).

Taken together, the results of our model fits indicate that (1) the structure of sensory processing pathways is not influenced by SCI (the Perceptual Competition model better accounted for both groups behavior), (2) differences in behavior for fast responses is the result of reduced stimulus salience in SCI (salience values for healthy controls > SCI), and (3) longer responses lead to increased perceptual competition in SCI.

## Discussion

Following our race model analyses using both the fastest (40%) and the entire RT distribution we found evidence of MSI deficits in participants with SCI. The audio-tactile condition was the most impaired one, as cross-modal inputs were not integrated in the SCI group. We believe that the audio-tactile condition exhibits the greatest sensory imbalance consistent with evidence showing somatosensory deficits^60,61^ and dominance of the auditory system in SCI^62,63^. For the visuo-tactile condition, we show that SCI participants can integrate cross-modal inputs, but to a lesser extent compared to control participants with a reduced integration across the 30th to 40th percentiles. In the audio-visual condition, although integration effects were reduced in the SCI group, comparisons between groups were not significantly different at any percentiles, indicating that audio-visual inputs may be normally integrated following SCI.

In contrast, results from the logistic fit on the entire distribution of RTs indicated deficits in MSI in the SCI group in all conditions. Specifically, differences in the intercept parameter (β0) between bimodal conditions and the race model across all stimulus-modalities were significantly lower in the SCI group compared to the control group (β0 value difference < 0 in the SCI group). That is, the MSI effect is lower in SCI participants compared to healthy controls and it decreases with increasing RTs.

These results are partially consistent with our results using only the fastest 40% of trials. However, we also noted some differences between these two approaches. Although MSI in the audio-visual condition appears unimpaired in the SCI group when considering only the fastest trials, SCI participants exhibit deficits in integrating stimuli in all three stimulus-modalities tested when considering the entire RT distribution. This may suggest that in healthy controls MSI effects persist over slower RTs across stimulus-modalities. In contrast, in individuals with SCI MSI effects are not only reduced but also decay with increasing RTs. Results from the entire RTs distribution are suggestive of sub-additive effects of integration in the SCI group. Sub-additive responses are observed when responses to multisensory stimuli are less than the summed unimodal responses^4^. Here we show that the multisensory effects are always super-additive in controls (i.e., responses to multisensory stimuli exceed the summed unimodal responses^4^), whereas sub-additive responses occur with increasing RTs during the processing of multisensory stimuli in participants with SCI.

Although our independent race model results demonstrate that MSI is impaired in SCI, they do not suggest specific mechanisms underlying this impairment. To address this question, we used a computational modeling approach and identified two mechanisms underlying MSI deficits after SCI. First, MSI deficits are due in part to specific unisensory differences between SCI and healthy controls. For SCI, both visual and tactile stimuli were less salient relative to healthy controls, while there was no difference between groups in the estimated salience of auditory stimuli. Thus, auditory processing appears to be less impacted by SCI than visual or tactile modalities during the processing of multisensory stimuli. The presence of one dominant modality can suppress other modalities, leading to a general reduced multisensory enhancement effect. Auditory information tends to be a dominant sensory modality in SCI^62,63^. For instance, auditory startle responses are faster in SCI participants compared to controls^62^ and salient auditory stimuli, are also discriminated faster in individuals with SCI compared to non-injured individuals^63^. Although participants with SCI were overall slower than healthy controls, our results are consistent with previous research suggesting that RTs to auditory stimuli are faster compared with visual stimuli. Our data indicate an effect of unisensory dominance. A dominance for auditory stimuli may underlie the salience deficits in MSI observed in our SCI group compared with healthy controls and relative to visual and tactile information.

To summarize, we provide evidence of a stimulus salience deficit in SCI during MSI. Normally our perception is facilitated by cross-modal information, and the stimuli are equally perceived. Following SCI, instead, the facilitation effect is impaired because there is an imbalance in perceiving cross-modal stimuli, because some stimuli are more or less salient than others. This deficit could be the result of a different way to perceive and interact with the environment when having an SCI.

We hypothesized that sensorimotor deficits in SCI would also impact MSI. In fact, MSI is strictly dependent on an intact sensorimotor system^41,42^. MSI is usually observed when cross-modal stimuli reduce the latency between sensory processing and motor commands, and thereby reduce sensory ambiguity^64^, help to plan a proper motor response^65^, and facilitate both perception and action^2,3^. The motor impairment caused by SCI may allow more time for stimuli to compete, resulting in multisensory depression. Our data support these assumptions showing that in a population in which the sensorimotor system is compromised, the principles of MSI fail with increasing RTs. It is possible that, following SCI, the inability to access the motor system within a certain time frame inhibits the integration process over time.

Indeed, when considering all trials our modeling results revealed that there was *increased competition* between perceptual representations in SCI for auditory, visual and tactile stimuli when combined with other modalities, i.e., the parameter estimates for those variables were higher, indicating more suppression of other modalities relative to healthy controls. In our SCI group, RTs were generally slower than for healthy controls in both unimodal and bimodal conditions, and application of the race model to the entire RT distribution revealed multisensory depression in SCI. Prolonged processing of multisensory information can lead to multisensory depression even in healthy controls, and responses to cross-modal stimuli are super-additive within a brief time-window^44–47^. Given this, it seems likely that the increased competition we observed in SCI is due to the overall slower processing of stimulus information rather than direct promotion of perceptual competition.

We have recently proposed a theoretical framework^37^ in which we argued that in SCI unisensory imbalance would contribute to multisensory depression and/or competition as opposed to integration. This is congruent with literature showing that an appropriate multisensory experience (i.e., the experience shaped by exposure to cross-modal stimuli^66^) and absence of unisensory imbalance is necessary for the integration to occur^3,16,20,39,40,67,68^. Our results largely support our proposed framework based on our modeling results. We observed that, for fast-response trials, salience for visual and tactile modalities was decreased in SCI participants relative to healthy controls, suggesting that auditory information dominated other modalities in our SCI group. Notably, increased competition between modalities was *not* observed during the fast trials. Instead, competition was observed only when considering all trials. The late appearance of competition in our SCI group suggests that initial unisensory imbalance leads to later increases in cross-modal competition. Although consistent with our theoretical framework, additional work is required to replicate and extend these findings.

Overall, we show that SCI undergoes MSI deficits involving different sensory modalities, we observe reduced super-additive effects, as well as sub-additive effects of MSI. These were associated with reduced stimulus salience and increased perceptual competition. Our work opens new research avenues for understanding and utilizing the neural mechanisms underlying MSI deficits to improve function after SCI. One promising avenue suggested by our results may be multisensory trainings (e.g., long term stimulation protocols using multisensory approaches) that can reduce cross-modal competition in SCI or prevent intact sensory modalities from suppressing other modalities. Such interventions may involve vision and auditory information, and even tactile information in incomplete injuries with residual sensorimotor pathways.

Furthermore, these findings are not only relevant to how MSI may be impaired by SCI, but also add to the existing literature on MSI in other population with nervous system trauma and non-clinical populations. Our model-based analyses of MSI data suggest a novel approach for quantifying behavioral performance during MSI in humans. By comparing formal fits of computational models instantiating multiple competing hypotheses, we found that perceptual competition best explained our behavioral data in both groups, highlighting a common mechanism underlying MSI in both healthy controls and SCI. Under information accumulation accounts, lateral competition between information accumulators is generally associated with cortical mechanisms involved in perception rather than merely sensory processing. While additional work is needed to replicate this finding, future work may focus more directly on how perceptual competition influences MSI in healthy individuals.

Finally, while we observed specific differences between SCI and healthy controls in stimulus salience and competition, this approach could be applied to other clinical populations that exhibit altered MSI as well. A reasonable possibility is that model fits to MSI data obtained from other clinical populations will reveal different patterns than those observed in this study. Although we observe decreased stimulus salience and increased perceptual competition in SCI, this pattern may underly MSI impairment in, e.g., schizophrenia, known to have MSI deficits associated to impaired ability to filter redundant sensory information, and unisensory abnormalities (e.g., visual or auditory hallucinations)^16^. Future work should examine whether and how the factors we identify in this study are influenced in other clinical populations.

## Methods

### Participants

Fifteen participants with SCI (mean age: 43.7 ± 12.6; Table 1) and seventeen healthy controls (mean age: 36.3 ± 7.8; *p* = 0.07) participated in the study. One participant with SCI (not included in final number of 15) was excluded due to missing responses (> 50%) in one of the experimental conditions (unimodal Tactile). To be enrolled in the study, participants had to have a chronic (≥ 1 yr), cervical or thoracic injury (C2–T10) (See Table 1). All participants gave informed consent to experimental procedures, which were approved by University of Miami Institutional Review Board (IRB) and conducted in accordance with the Declaration of Helsinki. All research was performed in accordance with relevant guidelines/regulations. All Participants had normal or corrected-to-normal vision, no history of hearing loss, psychiatric, or neurological trauma.

**Table 1:** Demographic and injury characteristics.

Subject	Age	Sex	Injury level	AIS	Etiology
1	44	M	C4	D	Tr
2	22	F	C4	D	Tr
3	60	M	C5	D	Tr
4	59	F	T1	C	Tr
5	61	M	C2	D	Tr
6	31	M	C5	A	Tr
7	37	M	C6	A	Tr
8	40	M	C3	D	Tr
9	59	M	C5	B	Tr
10	53	M	T6	A	Tr
11	47	M	C5	D	Tr
12	30	M	T4	A	Tr
13	33	M	C5	C	Tr
14	34	M	C5	B	Tr
15	46	M	C4	C	Tr

*AIS* American Spinal Injury Association Impairment Scale; *M* male; *F* female; *C* cervical; *T* thoracic; *Tr* traumatic.

Based on previous literature showing that multisensory effects are associated with medium and large effect sizes^69–71^, an a-priori sensitivity power analysis (G*Power 3 software^72^) revealed that our sample size was large enough to detect within–between interactions of interest in a mixed-design analyses of variance (ANOVA) which corresponds to a medium f = 0.3 effect size with a statistical power (1–β) of 0.95 (given α = 0.05, a correlation between repeated measures of 0.5, number of groups = 2, number of measurements = 6).

### Stimuli and procedure

The stimuli consisted of three unimodal stimulations (Auditory (A), Tactile (T) and Visual (V)) and their bimodal combination (AV, AT, VT). The A stimulus consisted of a pure tone (1000 Hz; 100 ms of duration) presented approximatively at 60 dB (measured with a sound meter from the participant's ear position) by means of headphones connected to E-prime Chronos, a USB-based multifunction response device^73^; the V stimulus consisted of a green letter “X” (1 cm; 34 pt Times New Roman font; 100 ms of duration) with a dark gray background presented in the middle of a computer screen (24″; resolution: 1920 × 1080; refresh rate: 60 Hz) at a viewing distance of approximatively 60 cm; the T stimulus consisted of a suprathreshold electrical simulation (duration of 200 µs) using a Digitimer (UK DS7A) delivered on the forehead (above level of injury for SCI participants). Individual thresholds of the tactile stimulus were set to be clearly suprathreshold using the method of the limits. Before to start the experiment, the intensity of the stimulator was set to 0 mA and then progressively increased by 1 mA until the subject reported to clearly perceive the stimulation. Then, the experimenter set the final stimulation at 20% of the perceived threshold and stimulated the subject for 10 times intermingled with 5 catch trials in which no stimulation was presented. The subject was asked to keep eyes closed and to report when he/she felt the tactile stimulus. If the subject did not detect 100% of the stimuli (i.e., if he/she failed to respond to some stimuli or gave false positives to the catch trials), the intensity was further increased by a 1 mA step, and the procedure was repeated.

The experiment consisted of a detection task (Fig. 1). Each trial started with a fixation cross (1500 ms) in the middle of a computer screen where participants were instructed to look, followed by a blank screen for a variable length of time (~ 500 -1500 ms) after which the target stimulus was presented. There were three types of stimuli that could be presented, either individually (unimodal trials: A, T, V) or paired—temporally congruent—(bimodal trials: AV, AT, VT), for a total of 6 conditions. Participants were instructed to respond vocally as fast as possible saying aloud “*yes*” when they perceived one of the unimodal or bimodal stimuli. After a variable ITI (~ 500–1500 ms) next trial started. Vocal reaction times (RTs) were collected using a microphone connected to E-prime Chronos, a USB-based multifunction response device^73^. The experiment consisted of a total of 360 trials (50 trials per condition and 60 catch trials in which no stimuli were presented) presented in random order and controlled by the E-Prime 3.0 software (Psychology Software Tools, Pittsburgh, PA). To prevent fatigue and maintain focus, participants were provided with a break every 25 trials. Before the main experiment, each participant also completed a training session, which consisted of 14 trials (2 trials for each unimodal and bimodal condition and 2 catch trials).

## Statistical analyses

### Raw RTs

Raw RTs were analyzed between groups and stimulus-modality. Three separate 2 × 3 between-subjects ANOVAs were performed with Group (SCI- healthy controls) as a categorial factor and stimulus-modality (A, V, AV); (V, T, VT) or (A, T, AT) as dependent variable.

### Test of the independent race model inequality (RMI)

Reaction times to multisensory stimuli are usually faster than reaction times to unisensory stimuli, as they provide synergistic information. This phenomenon is also known as redundant signals effect (RSE^74^)). Different models can be used to explain a RSE: race models and co-activation models^54,75^. Race models assume that the signal that is processed most rapidly is the signal that produces the response (i.e., the “winner” of the race). Co-activation models, on the other hand, assume that RT facilitation is accounted for by interactions that allow signals from concurrent information to combine non-linearly.

Race models, commonly implemented to examine multisensory effects, are robust probability (P) models that compare the cumulative distribution function (CDF) of combined unisensory reaction times, minus their product, to the CDF of multisensory reaction times. Hence, as a preliminary step, we tested the presence of MSI in our data that goes beyond RSE. RTs were sorted in ascending order by stimulus condition and then averaged on an individual basis^49^. For each participant, the RT range within the RTs was calculated across all the stimulus conditions and quantized into twenty bins from the fastest RT (or zero percentile) to the slowest RT (hundredth percentile) in 5% increments (0%, 5%, …, 95%, 100%). Differences between actual cumulative probability distributions [P (RTxy ≤ t)] and predicted cumulative probability distributions [min[P (RTx ≤ t) + P (RTy ≤ t) − P (RTx ≤ t) *P (RTy ≤ t)]] were calculated across each time bin and participant. Values greater than zero indicate a violation of the race model, suggestive of multisensory integrative processes. Significant violations of the race model were tested using Gondan’s permutation test over the fastest 40% of responses^48^. Differences in the magnitude of the race model violations were compared between groups in each stimulus-modality using simple effect analyses. False Discovery Rate (FDR) method^76^, to control for the rate of type I errors, was applied when necessary.

Additionally, we adopted a novel approach by analyzing race model violations across the entire RTs distribution, rather than considering only the fastest RTs up to 40% of the curve. We were interested in analyzing multisensory effects also for slower RTs to investigate whether such effects would persist or decline and whether they were similar or not in both groups, given that participants with SCI showed overall slower RTs compared with healthy controls. Raw RTs in each unimodal and the correspondent bimodal condition were first transformed to z-scores and then used to generate a set of empirical CDFs for each stimulus-modality. We then repeated the analysis described above on the standardized unimodal CDFs to generate the predicted RTs (race model), and a logistic fit was performed to obtain the coefficients of the logistic function (expressed as log(odds): β0 (intercept) and β1 (slope)) for each bimodal and race condition in each participant. Differences between bimodal conditions and the corresponding race in both coefficients were performed in each subject and stimulus-modality to obtain the final values of MSI effects. The difference values of the coefficients were then submitted to a 2 × 2 × 3 between-subjects ANOVA with Group (SCI- healthy controls) as a categorial factor and coefficients (β0-β1) and stimulus-modality (VT minus the corresponding Race, AT minus the corresponding Race, AV minus the corresponding Race) as dependent variables. A 2 × 3 within-subjects ANOVA was also performed in each group to check for differences between coefficients (β0-β1) and stimulus-modality (VT minus the corresponding Race, AT minus the corresponding Race, AV minus the corresponding Race).

### Computational models

To investigate the mechanisms underlying differences in reaction times and reaction time distributions, we used biologically inspired models of perceptual behavior based on temporally extended information integration^55,56^. Based on the results of the independent race model, we hypothesized that the observed differences between SCI and healthy controls could be the result of either changes in the structure of perceptual processing, or from changes in processing dynamics implemented on the same structure. To test whether the processing structure between SCI and healthy controls differed, we implemented two models. In the Stimulus Competition model (Fig. 6A), stimuli directly suppress the perceptual representation of cross-modal stimuli, while in the Perceptual Competition model (Fig. 6B), competition between stimulus modalities occurs indirectly via internal representations of stimuli. In both models, perceptual representations drive model output, equivalent to the “yes” response subjects were reporting when perceiving a stimulus.

Each model had 5 fixed parameters: weights from stimulus representations to perceptual representations were fixed at 1, as were the weights from perceptual representations to motor output. A response of the model was assumed when the activity of the output unit exceeded a fixed threshold of 1. Each model had 5 free parameters adjusted to fit the data. Two parameters (α) indicated stimulus “salience”, the capacity of a stimulus to drive activity in perceptual representation units. A noise parameter σ governed the variance of random, normally distributed noise added to the input of each unit in the model. Two competition parameters (β) determined the extent of inhibition of a perceptual unit activity, either due to stimulus salience from the paired modality (Stimulus Competition), or due to the activity of that modality’s perceptual representation (Perceptual Competition).

Both the Stimulus Competition and Perceptual Competition models consist of three layers of units. A stimulus layer with unit activity indicates the presence and strength of a stimulus for different sensory modalities. Activity in stimulus units propagates to perceptual representation units and correspond with the internal representation of a stimulus. Finally, a motor output unit is driven by activity in perceptual representation units, generating a response when a threshold is exceeded.

The activity of perceptual representation and motor output units is modeled as a leaky accumulator whose change *dx* in activity at each time step of a trial is described by the equation:

$$dx=I-x+N(0, \sigma )$$

Here, *I* is the sum of excitatory and inhibitory inputs to the unit, x is the unit’s current activity, and N () is Gaussian noise with zero mean and variance sigma.

For both models the net input *I* to the output unit was simply the sum of the activity of the perceptual representation units. Net input to perceptual representation units in both models was determined by the activity, *x*, of the associated stimulus modality and by cross-modal inhibition.

$${I}_{i}={{\alpha }_{i}x}_{i}-{{\beta }_{ji}c}_{j}$$

In the stimulus competition model, *c* is equal to the strength of the cross-modal stimulus. In the perceptual competition model, *c* is equal to the strength of the cross-modal perceptual representation. α indicates the stimulus salience for modality *i*, and *β* indicates the strength of cross-modal inhibition from modality *j* to *i.*

A single trial began with a bi- or unimodal stimulus being presented to the model and lasted until a response was generated (i.e., activity in the output unit exceeded threshold), or until 100 model iterations had elapsed. Following a response, activity in all units was set to 0 in preparation for the next trial. If, at any point in the trial, unit activity went below 0 due to random noise, its activity was set to 0 before the next model iteration.

Both models were fit to individual subject data (z-scored RTs) using an iterated grid search. For each of the 5 parameters, 5 initial values evenly distributed in the range 0.1 to 2 were tested, for a total of 3125 (5^5) unique parameter combinations. For each of these combinations, each model was simulated on 20 trials for each of the 3 bimodal and 3 unimodal conditions. As in our race model analyses, model RTs for pairs of unimodal conditions and the corresponding bimodal condition were z-scored. The distribution of z-scored model RTs was compared to the subject’s z-scored RT distribution (mean squared difference; MSD). The parameter combination with the lowest MSD was selected, and a new range of 5 values was established for each parameter. The new range was centered on the value of the parameter with the lowest MSD, and the total extent of the range was multiplied by 0.75, i.e., the next iteration of the grid search focused on a narrower range of values than the previous iteration. This iterative process was repeated a total of 10 times to generate best-fit values for each subject and each bimodal condition with its unimodal components. The iterated grid search was repeated 10 times, and the final best-fit value for each parameter was averaged over the 10 best-fit values generated by the iterated search. For each subject, we also recorded the average minimum MSD for all search iterations. As in our analyses using the race model, we fit our computational models both to all RTs as well as only to the fastest 40%.

## Data Availability

The datasets generated during and/or analysed during the current study are available from the corresponding author on request.

## References

[1] Meredith MA, Stein BE (1986). Visual, auditory, and somatosensory convergence on cells in superior colliculus results in multisensory integration. J. Neurophysiol..

[2] Stein BE, Meredith MA (1993). The merging of the senses xv, 211.

[3] Stein BE, Stanford TR (2008). Multisensory integration: Current issues from the perspective of the single neuron. Nat. Rev. Neurosci..

[4] Stevenson RA (2014). Identifying and quantifying multisensory integration: A tutorial review. Brain Topogr..

[5] Driver J, Noesselt T (2008). Multisensory interplay reveals crossmodal influences on ‘sensory-specific’ brain regions, neural responses, and judgments. Neuron.

[6] Noesselt T (2010). Sound-induced enhancement of low-intensity vision: Multisensory influences on human sensory-specific cortices and thalamic bodies relate to perceptual enhancement of visual detection sensitivity. J. Neurosci..

[7] Tyll S, Budinger E, Noesselt T (2011). Thalamic influences on multisensory integration. Commun. Integr. Biol..

[8] van den Brink RL (2014). Subcortical, modality-specific pathways contribute to multisensory processing in humans. Cereb. Cortex.

[9] Putzar L, Goerendt I, Lange K, Rösler F, Röder B (2007). Early visual deprivation impairs multisensory interactions in humans. Nat. Neurosci..

[10] Ro T, Wallace R, Hagedorn J, Farné A, Pienkos E (2004). Visual enhancing of tactile perception in the posterior parietal cortex. J. Cogn. Neurosci..

[11] Talsma D, Senkowski D, Soto-Faraco S, Woldorff MG (2010). The multifaceted interplay between attention and multisensory integration. Trends Cogn. Sci..

[12] Holmes NP, Spence C (2005). Multisensory integration: Space, time and superadditivity. Curr. Biol..

[13] Calvert GA, Thesen T (2004). Multisensory integration: Methodological approaches and emerging principles in the human brain. J. Physiol. Paris..

[14] Spence C, Driver J, Driver JC (2004). Crossmodal space and crossmodal attention.

[15] Macaluso E, Driver J (2005). Multisensory spatial interactions: A window onto functional integration in the human brain. Trends Neurosci..

[16] Williams LE, Light GA, Braff DL, Ramachandran VS (2010). Reduced multisensory integration in patients with schizophrenia on a target detection task. Neuropsychologia.

[17] Stekelenburg JJ, Maes JP, Van Gool AR, Sitskoorn M, Vroomen J (2013). Deficient multisensory integration in schizophrenia: An event-related potential study. Schizophr. Res..

[18] Hirst RJ (2020). The effect of eye disease, cataract surgery and hearing aid use on multisensory integration in ageing. Cortex.

[19] Stevenson R, Sheffield SW, Butera IM, Gifford RH, Wallace M (2017). Multisensory integration in cochlear implant recipients. Ear Hear..

[20] Guerreiro MJS, Putzar L, Röder B (2015). The effect of early visual deprivation on the neural bases of multisensory processing. Brain.

[21] Grunwald M, Weiss T, Assmann B, Ettrich C (2004). Stable asymmetric interhemispheric theta power in patients with anorexia nervosa during haptic perception even after weight gain: A longitudinal study. J. Clin. Exp. Neuropsychol..

[22] Gaudio S, Brooks SJ, Riva G (2014). Nonvisual multisensory impairment of body perception in anorexia nervosa: A systematic review of neuropsychological studies. PLoS One.

[23] Stevenson RA (2014). Evidence for diminished multisensory integration in autism spectrum disorders. J. Autism Dev. Disord..

[24] Feldman JI (2018). Audiovisual multisensory integration in individuals with autism spectrum disorder: A systematic review and meta-analysis. Neurosci. Biobehav. Rev..

[25] Foss-Feig JH (2010). An extended multisensory temporal binding window in autism spectrum disorders. Exp. Brain Res..

[26] Riva G, Gaudio S (2018). Locked to a wrong body: Eating disorders as the outcome of a primary disturbance in multisensory body integration. Conscious. Cogn..

[27] Freund P (2011). Disability, atrophy and cortical reorganization following spinal cord injury. Brain.

[28] Wrigley PJ (2009). Anatomical changes in human motor cortex and motor pathways following complete thoracic spinal cord injury. Cereb. Cortex.

[29] Turner JA, Lee JS, Schandler SL, Cohen MJ (2003). An fMRI investigation of hand representation in paraplegic humans. Neurorehabil. Neural Repair.

[30] Curt A (2002). Changes of non-affected upper limb cortical representation in paraplegic patients as assessed by fMRI. Brain.

[31] Henderson LA, Gustin SM, Macey PM, Wrigley PJ, Siddall PJ (2011). Functional reorganization of the brain in humans following spinal cord injury: Evidence for underlying changes in cortical anatomy. J. Neurosci..

[32] Ding Y, Kastin AJ, Pan W (2005). Neural plasticity after spinal cord injury. Curr. Pharm. Des..

[33] Chen R, Cohen LG, Hallett M (2002). Nervous system reorganization following injury. Neuroscience.

[34] Ghosh A (2010). Rewiring of hindlimb corticospinal neurons after spinal cord injury. Nat. Neurosci..

[35] Jacobs KM, Donoghue JP (1991). Reshaping the cortical motor map by unmasking latent intracortical connections. Science.

[36] Perani D (2001). Remodelling of sensorimotor maps in paraplegia: A functional magnetic resonance imaging study after a surgical nerve transfer. Neurosci. Lett..

[37] Vastano R, Costantini M, Widerstrom-Noga E (2021). Maladaptive reorganization following SCI: The role of body representation and multisensory integration. Prog. Neurobiol..

[38] Dambreville C (2019). Ankle proprioception during gait in individuals with incomplete spinal cord injury. Physiol. Rep..

[39] Yu L, Cuppini C, Xu J, Rowland BA, Stein BE (2019). Cross-modal competition: The default computation for multisensory processing. J. Neurosci..

[40] Otto TU, Dassy B, Mamassian P (2013). Principles of multisensory behavior. J. Neurosci..

[41] Gori M (2015). Multisensory integration and calibration in children and adults with and without sensory and motor disabilities. Multisens. Res..

[42] Gori M, Tinelli F, Sandini G, Cioni G, Burr D (2012). Impaired visual size-discrimination in children with movement disorders. Neuropsychologia.

[43] Huang S (2015). Multisensory competition is modulated by sensory pathway interactions with fronto-sensorimotor and default-mode network regions. J. Neurosci..

[44] Rowland BA, Quessy S, Stanford TR, Stein BE (2007). Multisensory integration shortens physiological response latencies. J. Neurosci..

[45] Meredith M, Nemitz J, Stein B (1987). Determinants of multisensory integration in superior colliculus neurons. I. Temporal factors. J. Neurosci..

[46] Chandrasekaran C (2017). Computational principles and models of multisensory integration. Curr. Opin. Neurobiol..

[47] Diederich A, Colonius H (2015). The time window of multisensory integration: Relating reaction times and judgments of temporal order. Psychol. Rev..

[48] Gondan M (2010). A permutation test for the race model inequality. Behav. Res. Methods.

[49] Mahoney JR, Verghese J (2019). Using the race model inequality to quantify behavioral multisensory integration effects. J. Vis. Exp. JoVE.

[50] Stevenson RA, Segers M, Ncube BL, Black KR, Bebko JM, Ferber S, Barense MD (2018). The cascading influence of multisensory processing on speech perception in autism. Autism..

[51] Scandola M (2019). Anticipation of wheelchair and rollerblade actions in spinal cord injured people, rollerbladers, and physiotherapists. PLoS One.

[52] Ulrich R, Miller J (1997). Tests of race models for reaction time in experiments with asynchronous redundant signals. J. Math. Psychol..

[53] Diederich A, Colonius H (2004). Bimodal and trimodal multisensory enhancement: Effects of stimulus onset and intensity on reaction time. Percept. Psychophys..

[54] Miller J (1982). Divided attention: Evidence for coactivation with redundant signals. Cognit. Psychol..

[55] Mazurek ME, Roitman JD, Ditterich J, Shadlen MN (2003). A role for neural integrators in perceptual decision making. Cereb. Cortex N. Y. N.

[56] Usher M, McClelland JL (2001). The time course of perceptual choice: The leaky, competing accumulator model. Psychol. Rev..

[57] Verbruggen F, Logan GD (2008). Response inhibition in the stop-signal paradigm. Trends Cogn. Sci..

[58] Bogacz R, Usher M, Zhang J, McClelland JL (2007). Extending a biologically inspired model of choice: Multi-alternatives, nonlinearity and value-based multidimensional choice. Philos. Trans. R. Soc. B Biol. Sci..

[59] Middlebrooks PG, Zandbelt BB, Logan GD, Palmeri TJ, Schall JD (2019). Countermanding perceptual decision-making. iScience.

[60] Jain N, Florence SL, Kaas JH (1998). Reorganization of somatosensory cortex after nerve and spinal cord injury. Physiology.

[61] Weber KA (2020). Assessing the spatial distribution of cervical spinal cord activity during tactile stimulation of the upper extremity in humans with functional magnetic resonance imaging. Neuroimage.

[62] Kumru H (2008). Exaggerated auditory startle responses in patients with spinal cord injury. J. Neurol..

[63] Pazzaglia M (2018). Embodying functionally relevant action sounds in patients with spinal cord injury. Sci. Rep..

[64] Green AM, Angelaki DE (2010). Multisensory integration: Resolving sensory ambiguities to build novel representations. Curr. Opin. Neurobiol..

[65] Stein BE, Rowland BA, Green AM, Chapman CE, Kalaska JF, Lepore F (2011). Chapter 10—Organization and plasticity in multisensory integration: early and late experience affects its governing principles. Progress in Brain Research.

[66] Stein BE, Stanford TR, Rowland BA (2014). Development of multisensory integration from the perspective of the individual neuron. Nat. Rev. Neurosci..

[67] Carriere BN (2007). Visual deprivation alters the development of cortical multisensory integration. J. Neurophysiol..

[68] Sinnett S, Soto-Faraco S, Spence C (2008). The co-occurrence of multisensory competition and facilitation. Acta Psychol. (Amst.).

[69] Barutchu A, Spence C (2020). An experimenter’s influence on motor enhancements: The effects of letter congruency and sensory switch-costs on multisensory integration. Front. Psychol..

[70] Tong J, Li L, Bruns P, Röder B (2020). Crossmodal associations modulate multisensory spatial integration. Atten. Percept. Psychophys..

[71] Villalonga MB, Sussman RF, Sekuler R (2021). Perceptual timing precision with vibrotactile, auditory, and multisensory stimuli. Atten. Percept. Psychophys..

[72] Faul F, Erdfelder E, Lang A-G, Buchner A (2007). G*power 3: A flexible statistical power analysis program for the social, behavioral, and biomedical sciences. Behav. Res. Methods.

[73] Babjack DL (2015). Reducing audio stimulus presentation latencies across studies, laboratories, and hardware and operating system configurations. Behav. Res. Methods.

[74] Hershenson M (1962). Reaction time as a measure of intersensory facilitation. J. Exp. Psychol..

[75] Miller J (1986). Timecourse of coactivation in bimodal divided attention. Percept. Psychophys..

[76] Benjamini Y, Hochberg Y (1995). Controlling the false discovery rate: A practical and powerful approach to multiple testing. J. R. Stat. Soc. Ser. B Methodol..

